# Design and evolution of a triad twisted string actuator for controlling a two degrees of freedom joint: improving performance and simulating active transmission adjustment

**DOI:** 10.3389/frobt.2026.1761507

**Published:** 2026-04-01

**Authors:** Damian Crosby, Joaquin Carrasco, William Heath, Lutong Li, Andrew Weightman

**Affiliations:** 1 Department of Mechanical and Aerospace Engineering, School of Engineering, Faculty of Science and Engineering, The University of Manchester, Manchester, United Kingdom; 2 Department of Electrical and Electronic Engineering, Faculty of Science and Engineering, The University of Manchester, Manchester, United Kingdom; 3 School of Computer Science and Engineering, College of Science and Engineering, Bangor University, Bangor, United Kingdom

**Keywords:** force control actuators, inverse dynamic control, robot joint module, snake robots, twisted string actuation system

## Abstract

Actuated universal joints are used in a wide range of robotic applications, including mobile snake robots, snake-arm robots and robotic tails. They are employed in applications such as search and rescue and confined space inspection. These can use remote cables, fluid driven systems, or inline motors. To realise the benefits of inline actuation while keeping the system compact with a high power to weight ratio, an actuated universal joint (AUJ) was developed using an ‘‘antagonistic triad’’ of three twisted string actuators in our previous work. However, the design had numerous drawbacks in its prototype form, namely, a limited angle range, poor accuracy due to the angular feedback sensors used, and issues with string failure due to mechanical design choices. In this publication, we performed a root-cause analysis of these issues, and partially or fully mitigated some of them by reducing the distance between the twisted string actuator (TSA), removing geometry which caused premature string failure, and exchanging the angular feedback sensors for more accurate ones. As a result, angle range was increased from ± 14.5° to ± 26° for a single axis, and ± 6° to ± 20° for a dual axis movement. Angular feedback sensor accuracy increased from ± 0.21° to ± 0.11°, and no string failures occurred within load limits. The performance of the mechanism was further characterised with additional experiments for increased follower load and angular velocity. A novel method to adjust the transmission ratio during operation (active transmission adjustment) was proposed and simulated, and its advantages over existing mechanisms for a snake robot in a multi-segment configuration were theoretically evaluated.

## 1 Introduction

Actuated universal joint (AUJ) mechanisms are crucial in various robotic applications like confined space inspection with continuum robots, highly manoeuvrable snake robots, and biomimetic robot tails for stability. These mechanisms can either use inline actuators, which directly move the joint (Rezaei et al., 2008; Baba et al., 2010; Wright et al., 2012), or cable/fluid driven systems relying on a static ‘‘base’’ to house actuators or compressors (Buckingham and Graham, 2012; Rone et al., 2018; Qin et al., 2023). Inline actuators necessitate high torque due to lifting the mass of subsequent sections, while systems with a static base require additional space, limiting their utility in mobile robots.

First developed by Würtz et al. (2010), the twisted string actuator (TSA) uses two or more strings between two fixtures as a linear actuator. Rotating one fixture, typically with an electric motor, twists the strings into a helix, decreasing the distance between them. TSAs have applications in hand orthoses, elbow joints, foldable robot arms, and more (Muehlbauer et al., 2021; Park et al., 2020; Suthar and Jung, 2021).

Alternative electric actuation systems, like leadscrews, require additional gearing for significant reduction increases, which results in larger and heavier actuators. In contrast, a TSA can achieve reduction increases by reducing string thickness or count, thus slightly reducing actuator mass (Palli et al., 2012). While leadscrews can marginally increase reduction by decreasing thread lead (Budynas and Nisbett, 2011), this approach faces challenges related to manufacturing tolerances and material limitations. Increasing screw radius also enlarges actuator size and mass similarly. Therefore, from a mechanical perspective, TSA holds an advantage over leadscrews by increasing reduction without adding mass.

One of TSAs main challenges is its reduction variation with motor angle, exhibiting an inverse nonlinear relationship (Würtz et al., 2010; Palli et al., 2012). String compliance under high force conditions also requires consideration, which can be addressed through accurate modelling (Nedelchev et al., 2020) or a robust control strategy that disregards compliance in the system model (Würtz et al., 2010; Palli et al., 2012). Since TSA can only apply force in tension due to string flexibility, single DOF TSAs joints need a spring return mechanism, limiting actuator range as spring force increases with decreasing maximum TSA force (Usman et al., 2017b). A linear force return mechanism was developed to partially mitigate this issue, but the ideal solution involves using a second synchronized TSAs to achieve a matching antagonistic force profile (Popov et al., 2013; Park et al., 2016; Lee et al., 2019; Park et al., 2020). By adding a third TSA, a 2 DOF joint can be created, as demonstrated by similar cable driven systems (Dessen, 1986).

In Crosby et al. (2022), we developed an initial proof of concept and demonstrated robust closed-loop control of this mechanism though experimental validation.

The use of TSAs as an actuator for AUJs remains an understudied area of research. Konda et al. (2023) proposed a similar design using a flexible core with continuous curvature instead of a rigid universal joint, presenting an open-loop control solution for multi-axis control using only two TSAs at a time, albeit with a limited azimuthal axis range. In contrast, we demonstrated closed-loop control in both axes of motion, covering the full azimuthal range of $\left.\left[0,360\right.\right)$ °, using three TSAs in an ‘‘antagonistic triad’’ configuration. This results in a light, compact AUJ design with potential improvements in power to weight ratio over existing inline actuation options, such as direct drive motors or leadscrew actuators.

Our aim in this publication is to address the issues present in the original design and analyse and evaluate the performance characteristics and special properties of the TSA antagonistic triad mechanism, in order to prepare it for implementation in a future practical application. We extended our work in Crosby et al. (2022) by identifying shortcomings in the original prototype (hereafter referred to as such) and addressing them through design changes to create an ‘‘improved prototype’’ (hereafter referred to as such). We also conducted supplemental experiments to further characterise the mechanism, by examining the effect of additional follower mass and increased joint velocity. Finally, we have looked at existing snake robots in literature, and have considered how a theoretical robot with this mechanism would compare in terms of performance, specifically the self-supporting segment limit. The main contributions of this work are as follows:

- Identification of improvements that could be made to the original prototype through design changes, and experimental results detailing the performance improvements realised as a consequence.
- Experiments conducted to further characterise the performance of the AUJ by the addition of mass to the follower and increasing joint velocity.
- A simulation of ATA which allows the mechanism to change specific performance characteristics in real time during operation.
- Analysis of a theoretical multi-segment antagonistic triad TSA design against existing snake robots.

In Section 3 we give a brief summary of the design of the original prototype, in order to furnish the reader with enough information to understand the rest of the publication. More information can be found in Crosby et al. (2022). In Section 4 we identify each improvement we address through design alterations, explain the alterations made, and show the results that demonstrate the improved performance. In Section 5 we show the results of additional experiments to further characterise the mechanism. In Section 6 we discuss the remaining limitations of the improved prototype, the ATA simulation, and a distributed control proposal for multi-segment operation. We also compare a theoretical multi-segment design to other snake robots in this section. Finally, Section 7 then summarises the publication and discusses future work.

## 2 TSA background

**FIGURE 1:**
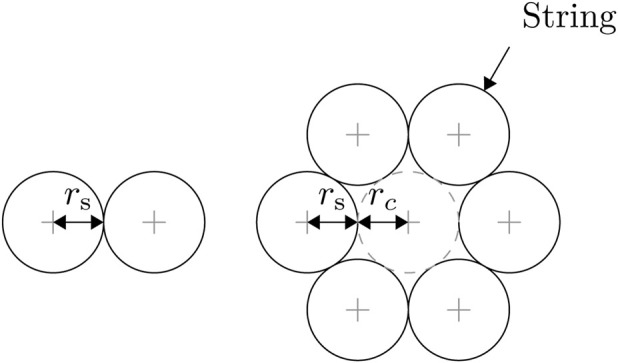
The location of $r_{\text{s}}$ and optionally $r_{c}$ in a string bundle.

**FIGURE 2:**
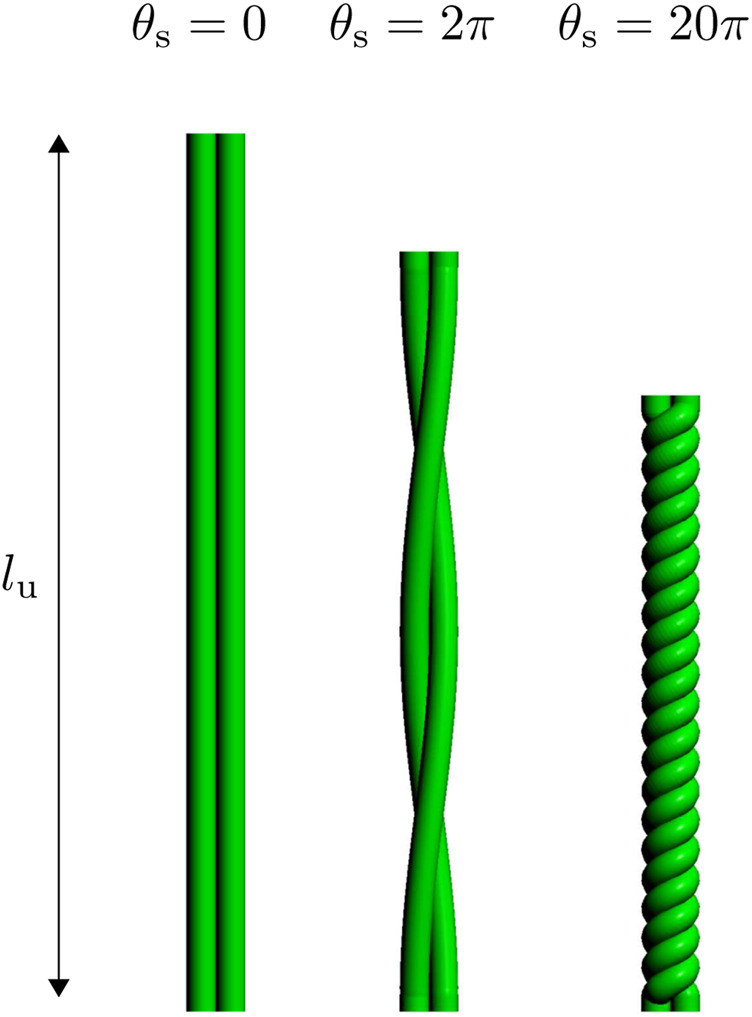
The value of $\theta_{\text{s}}$ increases the number of twists in a string bundle with a string length $l_{\text{u}}$ .

Given the unwound length $l_{\text{u}}$ in meters, and the cross-section radius of the string $r_{\text{s}}$ (or $r_{\text{s}}$ + $r_{c}$ when there are more than two strings, where $r_{c}$ is the radius of a tangentially constrained circle drawn between the strings) in meters as shown in Figure 1. Equation 1 gives the actuator length in metres as:

$$l_{\text{s}}\left(\theta_{\text{s}}\right)=\sqrt{l_{\text{u}}^{2}-\theta_{\text{s}}^{2}r_{\text{s}}^{2}},$$ (1)

where $\theta_{\text{s}}$ is the motor angle in radians, as shown in Figure 2.

Although theoretically the stroke of the TSA can be the entire domain of $\left[0,l_{\text{u}}\right]$ , in reality the thickness of the string prevents a geometric helix from forming once the helix pitch $q<4r_{\text{s}}$ (or $q<2nr_{\text{s}}$ for n strings). This is defined by Equation 2 which limits the lower bound of the stroke as follows,

$$l_{\min}=\frac{l_{\text{u}}}{\sqrt{\frac{\pi^{2}}{2}+1}}\approx 0.46\;l_{\text{u}}$$ (2)

or approximately 46% of $l_{\text{u}}$ for a two string TSA.

## 3 Original prototype design summary

**FIGURE 3:**
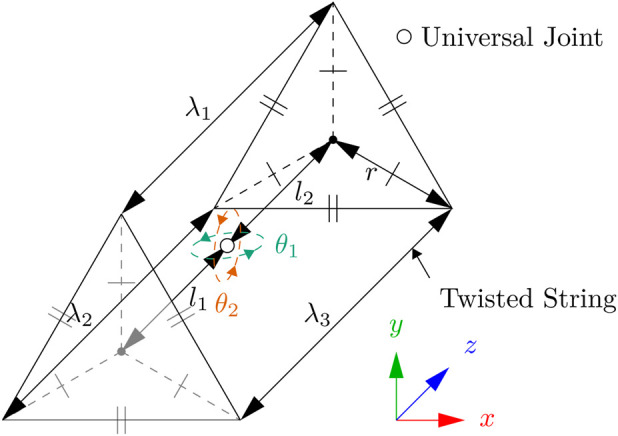
Kinematic diagram of the antagonistic triad, where the universal joint rotation is defined by $\theta_{1,2}$ on the y and x axes respectively, and the actuator lengths are defined by $\lambda_{1,2,3}$ for the *top*, *left* and *right* strings, and r and $l_{1,2}$ define the anchor points of the strings.

Because TSA can only operate in tension, a minimum of three TSAs are required to operate a AUJ. These can be arranged into an ‘‘Antagonistic Triad’’ where adjusting the length of each TSA changes the orientation of the AUJ, similarly to other cable driven robotic systems (Qin et al., 2023). These lengths can be combined into Equation 3, a vector function:

$$\begin{array}{cc} \Lambda \left(\theta \right) & =\left[\begin{array}{ccc} \lambda_{1}\left(\theta \right) & \lambda_{2}\left(\theta \right) & \lambda_{3}\left(\theta \right) \end{array}\right]\\ \lambda_{1}\left(\theta \right) & =\sqrt{\begin{array}{cc} a & +2l_{1}r\sin\left(\theta_{2}\right)\cos\left(\theta_{1}\right)+l_{2}^{2}\\ & +2l_{2}r\sin\left(\theta_{2}\right)-2r^{2}\cos\left(\theta_{2}\right)+2r^{2} \end{array}}\\ \lambda_{2}\left(\theta \right) & =\sqrt{a+b+c-d}\\ \lambda_{3}\left(\theta \right) & =\sqrt{a-b-c+d}\\ \text{where:}\\ a & =l_{1}^{2}+2l_{1}l_{2}\cos\left(\theta_{1}\right)\cos\left(\theta_{2}\right)\\ b & =\sqrt{3}l_{1}r\sin\left(\theta_{1}\right)-l_{1}r\sin\left(\theta_{2}\right)\cos\left(\theta_{1}\right)+l_{2}^{2}\\ c & =\sqrt{3}l_{2}r\sin\left(\theta_{1}\right)\cos\left(\theta_{2}\right)-l_{2}r\sin\left(\theta_{2}\right)\\ d & =\frac{\sqrt{3}r^{2}\sin\left(\theta_{1}\right)\sin\left(\theta_{2}\right)}{2}-\frac{3r^{2}\cos\left(\theta_{1}\right)}{2}-\frac{r^{2}\cos\left(\theta_{2}\right)}{2}+2r^{2}, \end{array}$$ (3)

where Λ(θ) is a vector function which outputs the magnitudes of each point pair. This function also represents the lengths of each actuator, assuming both ends of each actuator can rotate freely on both x and y axes. Other coefficients are labelled in Figure 3.

After an investigation into a position-based kinematic control system in the original prototype proved unsuccessful, a force-based control system was developed, which uses the inverse dynamics of the AUJ to convert angular velocity from a PID controller into angular torque, which is then turned into force setpoints for each TSA using an optimising algorithm based on Dessen (1986). The control system is a four layer cascade design, joining an outer loop PID controller $C_{1}$ with coefficients $k_{\text{p}}$ , $k_{\text{i}}$ and $k_{\text{d}}$ to an inverse dynamic control system $C_{2}$ (Spong et al., 2020), to the triad force controller $C_{3}$ in Dessen (1986), to a proportional controller $C_{4}$ with coefficient $k_{p_{\text{s}}}$ for each TSA. It uses feedback signals of the joint position and TSA force.

$C_{3}$ uses the *inverse force transformation* optimisation algorithm from Dessen (1986) with the jacobian of Equation 3 to select an optimal force vector from the desired joint torque. Equation 4 presents this in an unexpanded and more general form,

$$\begin{array}{cc} J_{\Lambda } & =\left[\begin{array}{ccc} \frac{\partial \lambda_{1}}{\partial \theta_{1}} & \frac{\partial \lambda_{2}}{\partial \theta_{1}} & \frac{\partial \lambda_{3}}{\partial \theta_{1}}\\ \frac{\partial \lambda_{1}}{\partial \theta_{2}} & \frac{\partial \lambda_{2}}{\partial \theta_{2}} & \frac{\partial \lambda_{3}}{\partial \theta_{2}} \end{array}\right]\\ \gamma \left(i\right) & =-J_{\Lambda_{-i,*}}^{-⊺}\left(J_{\Lambda_{i,*}}^{⊺}f_{\min}+\tau \right)\\ F\left(\tau ,\theta \right) & =\left[\begin{array}{ccc} f_{\min} & \gamma {\left(2\right)}_{1} & \gamma {\left(3\right)}_{1}\\ \gamma {\left(1\right)}_{1} & f_{\min} & \gamma {\left(3\right)}_{2}\\ \gamma {\left(1\right)}_{2} & \gamma {\left(2\right)}_{2} & f_{\min} \end{array}\right]. \end{array}$$ (4)

A force matrix F is created from torque input τ , jacobian $J_{\Lambda }$ from vector function $\Lambda \left(\theta \right)$ as defined in Equation 3, and minimum force constant $f_{\min}$ . $f_{ii}$ is equal to $f_{\min}$ , while the other elements in the column are based on a calculation using $J_{\Lambda_{-i,*}}$ where −i is a row removed from the matrix. Algorithm 1 is then used to create output force vector $f_{\text{set}}$ , which minimises the net force on all TSA while producing the desired output torque on the universal joint. This algorithm is implemented directly rather than using a general linear programming solver in order to improve performance in a real-time system.

Algorithm 1 Selects one column of F to be the output force vector $f_{\text{set}}$ , where ⊤ and ⊥ are boolean true and false respectively, s is a vector of three booleans, and $f_{*,i}$ is the ith column of F .

1: $s\leftarrow \left[\begin{array}{ccc} ⊤ & ⊤ & ⊤ \end{array}\right]$

2:  **if** $f_{23}>f_{\min}$ **then** $s_{2}\leftarrow ⊥$ **else** $s_{3}\leftarrow ⊥$ **end if**
3:  **if** $f_{31}>f_{\min}$ **then** $s_{3}\leftarrow ⊥$ **else** $s_{1}\leftarrow ⊥$ **end if**
4:  **if** $f_{12}\geq f_{\min}$ **then** $s_{1}\leftarrow ⊥$ **else** $s_{2}\leftarrow ⊥$ **end if**
5:  **for** i=1 to 3 **do**
6:    **if** $s_{i}\to ⊤$ **then** $f_{\text{set}}\leftarrow f_{*,i}$ **end if**
7:  **end for**

**FIGURE 4:**
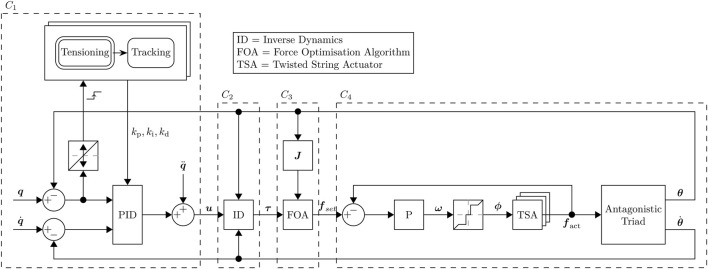
Block diagram of the complete experimental control system, excluding the hardware velocity controllers for the motors. Dashed boxes correspond to the control layers $C_{1\;\ldots 4}$ .

**FIGURE 5:**
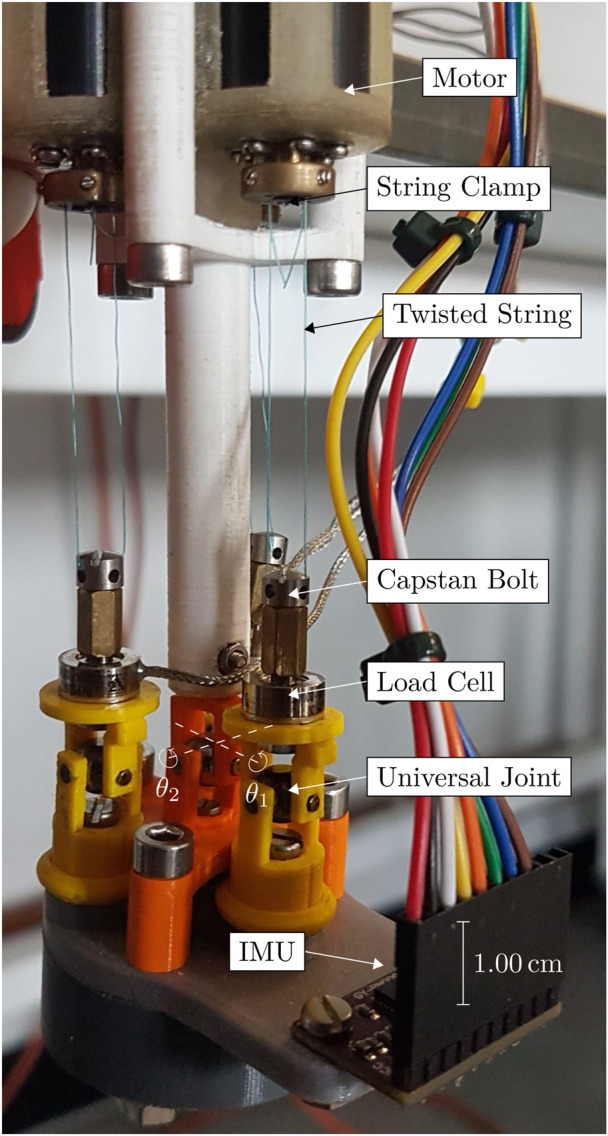
Annotated photograph of the original prototype, with the roll $\theta_{1}$ and pitch $\theta_{2}$ axes marked.

The mechanism starts off with all TSA fully unwound, with $f_{\text{act}}\approx {\left[\begin{array}{ccc} 0 & 0 & 0 \end{array}\right]}^{⊺}$ , so there is large step response from the motors to reach the initial force setpoints for string tension. This is called the ‘‘tensioning’’ state, which uses different PID coefficients (which are in Table 1) in order to shorten the startup time required for the mechanism. The control system then transitions to the ‘‘tracking’’ state on a per-axis basis, when there is a zero crossing, which allows the mechanism to settle at $\theta ={\left[\begin{array}{cc} 0 & 0 \end{array}\right]}^{⊺}$ . As the TSA are hand assembled, there are slight variations in $l_{\text{u}}$ which the PID controller must compensate for. To prevent the TSA from becoming ‘‘over twisted’’ (Palli et al., 2012; Crosby et al., 2022), the motor angles are monitored during operation, and the mechanism resets if it exceeds the twist limit as defined in Equation 2. Since the three load cells used to measure $f_{\text{act}}$ are connected to the follower body via universal joints (Crosby et al., 2022), the TSA tension force is always coaxial to the load cell axis, and any perpendicular forces can be assumed to be negligible. The main control loop was operated at a frequency of 5 Hz. Figure 4 shows a complete block diagram of the control system, and Figure 5 is an annotated photograph of the original prototype.

**TABLE 1:** PID gains used for the experiment.

Gain	Value	
	Tensioning	Tracking
$k_{\text{p}}$	$2.00\times 10^{5}$	$8.00\times 10^{5}$
$k_{\text{i}}$	3500.00	3500.00
$k_{\text{d}}$	0.00	50.00
$k_{{\text{p}}_{\text{s}}}$	5.00	5.00

### 3.1 Original prototype limitations

**FIGURE 6:**
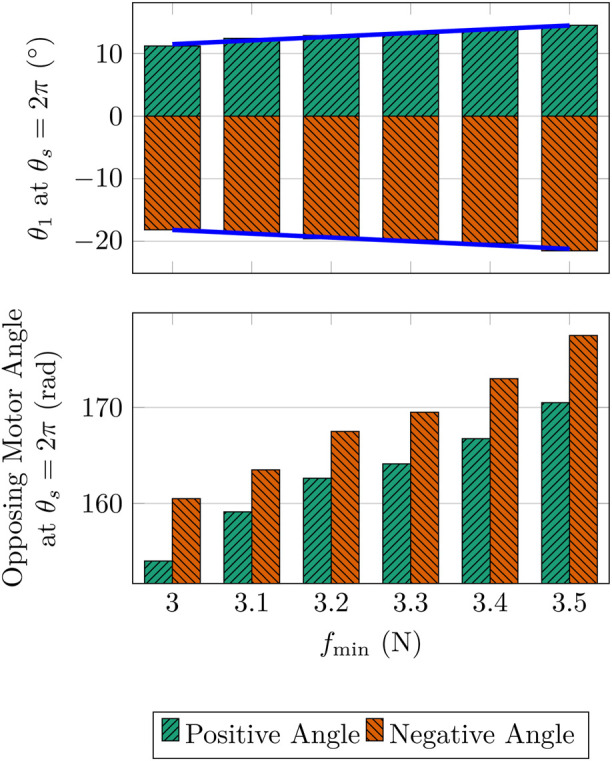
AUJ roll angle $\left(\theta_{1}\right)$ of the original prototype when the smallest TSA motor angle is equal to 2π , and motor angle $\left(\theta_{\text{s}}\right)$ of the ‘‘opposing’’ TSA (the TSA with the largest motor angle) at the same position.

After the experiments were conducted on the original prototype in Crosby et al. (2022), several shortcomings with the design were noted that reduced the performance of the system:

- Limited AUJ Angle Range - The AUJ angle tracking experiments in Crosby et al. (2022) only had a range of ± 14.5° in a single axis, and ± 6° for both axes. This was because one or more TSA would completely ‘‘unwind’’ near that limit and be unable to lengthen further. This limits the practicality of such a mechanism, for example, a multi-segment design would have a very large minimum curvature. Increasing $f_{\min}$ does increase the angle range marginally by increasing the TSA motor angle at $\theta ={\left[\begin{array}{cc} 0 & 0 \end{array}\right]}^{⊺}$ , which was proven experimentally, as shown by experiments conducted on the original prototype in Figure 6. These experiments were able to achieve modest increases of 5.97° N^−1^ for the positive (upper) limit of the universal joint roll $\theta_{1}$ , and 6.07° N^−1^ for the negative (lower) limit, within the $f_{\min}$ interval $\left[3,3.5\right]$ . However, further attempts to increase $f_{\min}$ , or attempts to perform the same experiment on the pitch axis resulted in premature string failure. It was also clear to achieve significant increases in AUJ angle range, a different approach would be needed that would require modifications to the design.
- Poor AUJ Angle Measurement Accuracy - The original prototype used a Bosch Sensortec BNO080 9 DOF inertial measurement unit (IMU) (BNO080 Data Sheet, 2017) to measure the AUJ orientation. The original plan was to use two IMUs on the base and follower bodies, and the orientation would be calculated from the difference. However, the magnetometer measurements proved to be unreliable inside the laboratory, so only a single IMU was used, and the base segment was orientated with the gravity vector parallel to the z axis. This allowed the AUJ orientation to be calculated from only the accelerometer readings, but meant the mechanism could only be controlled when orientated in the vertical axis. There was also an issue with the IMU resolution, which was only accurate to within ± 1.5° (BNO080 Data Sheet, 2017). A Savitsky-Golay filter (m=11,k=1) was applied to the results in Crosby et al. (2022) for data presentation purposes and to more accurately represent the true AUJ angles at that point in time.

Also, to enable the mechanism to be controlled in any direction relative to gravity, a solution that can directly measure the mechanical angular displacement of the universal joint was required.

- String Failures - One reoccurring issue was the TSA strings breaking at high values of $\theta_{\text{s}}$ , which became more common after a number of twisting and untwisting cycles. Occasionally this was caused by a failure of limit monitoring within the control system when $f_{\text{act}}>9$ which could be attributed to tensile failure of the string, but failure would also occur when $f_{\text{act}}\leq 9$ , which would have been for other reasons, such as wear. This was caused by wear on the strings generated by ‘‘pinch points’’ and ‘‘bite points’’ in the mechanism geometry as shown in Figure 8a.

## 4 Methodology

### 4.1 Original prototype improvements

#### 4.1.1 Increasing AUJ angle range

The most effective modification to improve AUJ angle range is to reduce the value of r in (Equation 3). This decreases the stroke length required for each TSA to achieve the same AUJ angle range (as shown by Table 2), which also reduces the TSA motor angle accordingly, allowing a greater AUJ angle to be reached before one or more TSA unwind. One issue is that reducing r also reduces the maximum motor cross-section if the motor shafts remain co-axial with the TSA strings. The simplest solution is to use smaller motors, but the same size or larger motors can be used if the design is changed to include offset shafts connected by spur gears. Smaller motors were chosen, with a cross-section of only 120 mm^2^ compared to 227 mm^2^ on the existing design. These were Guangdong Kingly Gear Co. Micro Metal Gearmotors (50:1) (JL-12FN20-50-06420, (2020), which were lighter than the existing motors (18 g compared to 27 g), and the mechanism would be less complex. These motors have a much lower ${\dot{\theta }}_{\max}$ than the existing motors (44 rads^−1^ compared to 442 rads^−1^ (DC Micromotors - Metal Commutation, 2020). Since the original experiments limited the motor velocity to 10rads^−1^ to ensure mechanism stability, this is not a concern. This change of motors allowed r to be reduced from 13 mm to 7.25 mm.

**TABLE 2:** Table of the minimum and maximum values of Λ(θ) at different values of r in the interval of $\left[-\frac{\pi }{2},\frac{\pi }{2}\right]$ .

r [mm]	$\lambda_{1}$ [mm]		$\lambda_{2}$ [mm]		$\lambda_{3}$ [mm]	
	Min	Max	Min	Max	Min	Max
13.0	42.64	68.35	42.64	68.57	42.64	68.57
10.0	45.02	64.88	45.02	64.98	45.02	64.98
7.25	47.33	61.78	47.33	61.82	47.33	61.82

**FIGURE 7:**
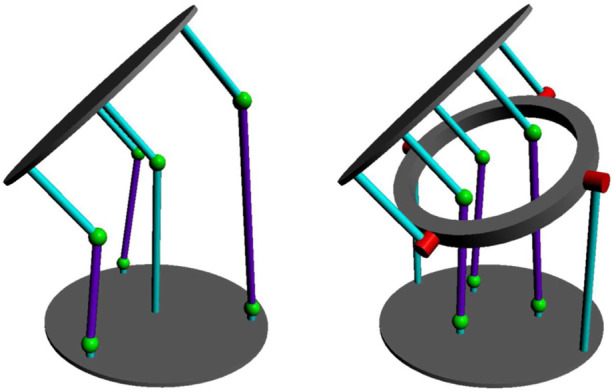
An AUJ with a central universal joint, and one with a hollow spider. The hollow spider allows r to be decreased as space is no longer required for a central universal joint.

To achieve this reduction, additional design changes were necessary. The proximity of the TSA strings made a central shaft impractical, unlike in the original prototype. Instead, a ‘‘hollow spider’’ arrangement was adopted for the universal joint, allowing the TSA strings to pass through its centre, as shown in Figure 7. This modification also provided space for directly installing AUJ angle sensors onto the universal joint, as detailed in Section 4.1.2.

#### 4.1.2 Improving AUJ angle measurement accuracy

Two options were considered to address this issue: potentiometers and hall effect sensors. A potentiometer would link each universal joint spider axis to a resistive track, altering its resistance based on AUJ orientation and providing an analogue voltage signal to the controller. Mechanical stops would ensure the required AUJ angle range could be measured during assembly. Alternatively, a hall effect sensor would use a radially bipolar magnet on the shaft’s end with a hall effect sensor beneath it, providing analogue voltage signal or digital data for the axial orientation of the magnet and, consequently, the shaft (Bienczyk, 2009). The potentiometer solution, chosen for its simplicity of control integration and integrated mechanical stops, would be simpler to integrate into the existing control system, requiring only a (ADC) and a slope-intercept form for conversion to a AUJ angle. The Bourns PDB08, selected for its small size and internally threaded shaft, was chosen as the potentiometer (PDB08 - 8 mm Micro Rotary Potentiometer, 2021). Operating as a voltage divider at + 5 V, the PDB08 would offer a resolution of approximately 40° V^−1^. With the 12-bit resolution of the onboard ADC for myRIO analogue inputs, this yields a theoretical resolution of approximately 0.05° compared to ± 1.5° for the BNO080. The potentiometer’s sliding noise (maximum 100 mV) would reduce this during AUJ motion, partially mitigated with a 64:1 oversampling filter. This improvement from the IMU was deemed significant enough for use in the latest prototype, validated through experimental testing, rendering further investigation of the hall effect sensor unnecessary.

#### 4.1.3 Preventing string failure

**FIGURE 8:**
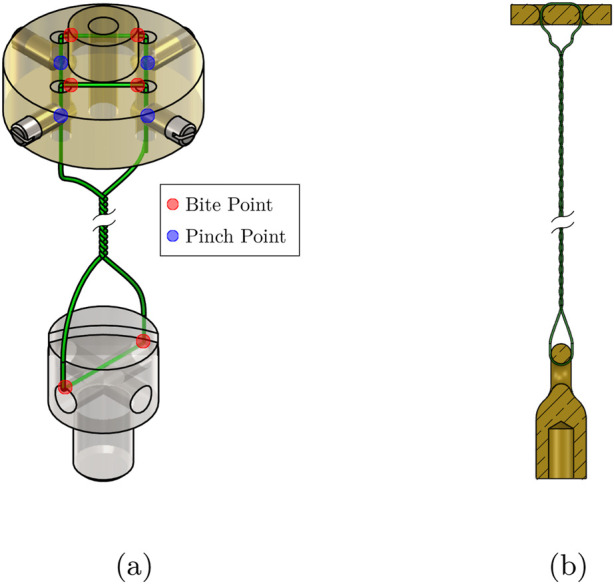
**(a)** The original TSA string loop between the string clamp and capstan bolt, with ‘‘pinch’’ and ‘‘bite’’ points indicated, where premature string failure may occur. **(b)** Section view of the new TSA string loop showing how the pinch and bite points have been eliminated.

Analysis of the design revealed ‘‘pinch points’’ and ‘‘bite points’’, where the string could deform or weaken due to contact with surfaces or sharp edges, as depicted in Figure 8a. This is similar to the observations made in Usman et al. (2017a). Removing these points by rounding off edges and using alternative string securing methods, such as tying it into a loop instead of using grub screws, eliminated potential sources of string damage, as shown in Figure 8b. However, nylon monofilament, like the TSA string, is still prone to torsion fatigue (Goswami and Hearle, 1976; Toney and Schwartz, 1992), reducing its tensile strength. To address this, the SeaKnight BLADE 0.2 mm nylon monofilament was replaced with 0.2 mm Dyneema® polyfilament string, as used in Palli et al. (2012). A lifecycle analysis of this material was conducted in Palli et al. (2012), Usman et al. (2017a), under various loads and stroke lengths, and we consider these experiments sufficiently rigorous as to not require replication in our specific implementation. Design changes to the string clamp facilitated easier installation of the polyfilament string, making it a practical option.

#### 4.1.4 Final design of improved prototype

**FIGURE 9:**
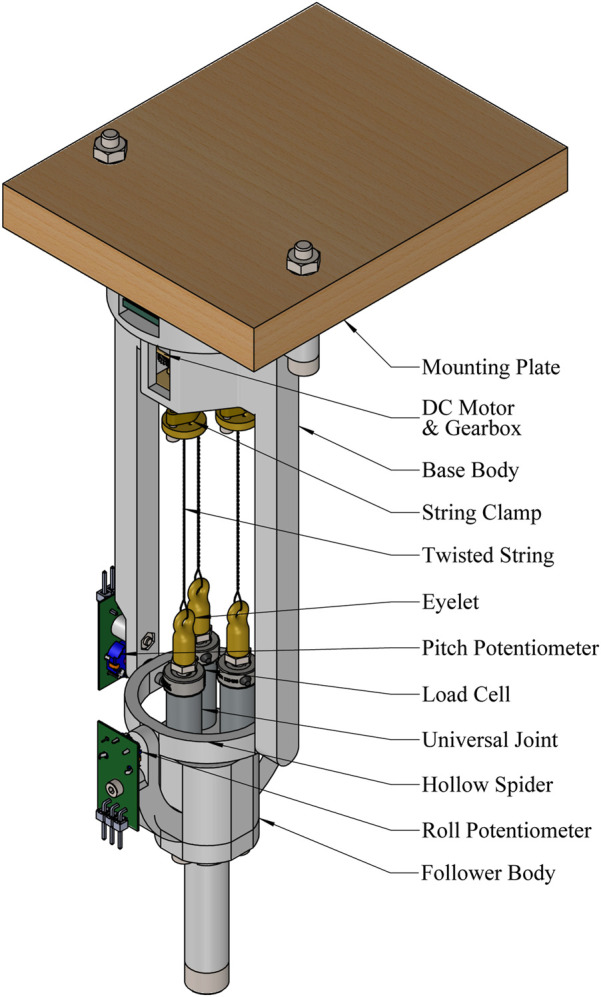
Schematic of the latest prototype with labelled components.

**FIGURE 10:**
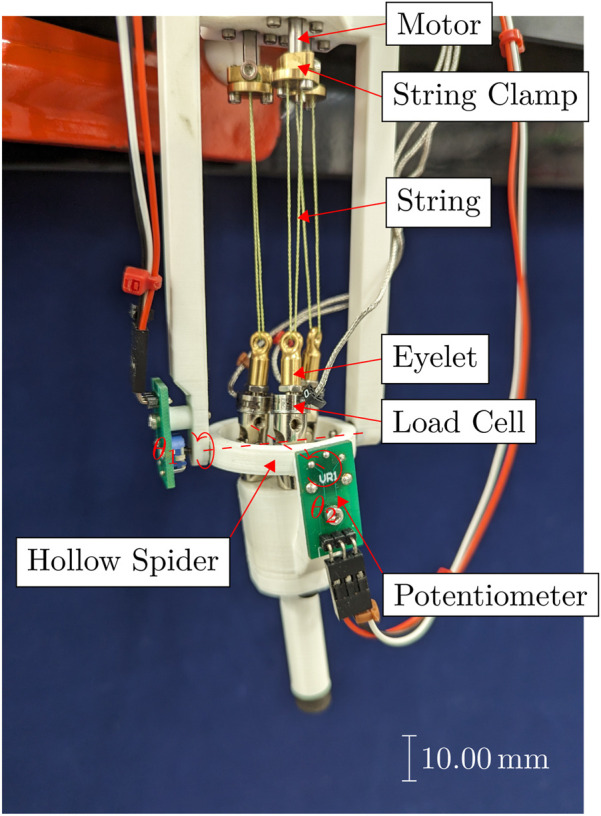
Annotated photograph of the single segment physical prototype antagonistic triad, with the roll $\theta_{1}$ and pitch $\theta_{2}$ axes marked.

Figure 9 presents an annotated schematic of the improved prototype, and Figure 10 shows a photograph with the AUJ axes annotated. The load cells remained unchanged from the original prototype, while alterations were made to the universal joints below them. The hollow spider was connected to the base and follower bodies using machine precision bearings and bushings to ensure smooth motion. Regarding the control system, the Faulhaber MCDC3002 motor controllers (Motion Controllers - Series MCDC 3002 S, 2018) were replaced with a single Pimoroni Motor 2040, capable of handling up to four Micro Metal Gearmotors with Micro Metal Motor Encoders attached. Programming was done with a velocity controller similar to the one provided in the pimoroni-pico library. Furthermore, the deadband compensation (used to reduce small motor motions (Crosby et al., 2022) threshold was reduced to ± 0.5 rad s^−1^ due to the smaller deadband region of the motors, while the motor velocity limit of 10 rad s^−1^ remained unchanged. The deadband compensation could be increased if energy consumption is an issue, at the expense of increased tracking error and insensitivity to small setpoint changes. Table 3 shows the model coefficients for the improved prototype.

**TABLE 3:** Model coefficients for the latest prototype.

Coeff.	Value	Coeff.	Value
$l_{1}$	55.00 mm	$f_{\min}$	3.00 N
$l_{2}$	0.00 mm	$r_{\text{s}}$	100.00 µm
r	7.25 mm	m	61.60 g
$l_{\text{u}}$	55.00 mm	${\dot{\theta }}_{s_{\max}}$	44.00 rad s^−1^
$\tau_{\max}$	0.04 N m	ρ	$\left[\begin{array}{ccc} 0.00 & 0.00 & 3.05 \end{array}\right]$ mm

Coeff.	Value				
I	$\left[\begin{array}{ccc} 2.8\times 10^{-5} & 0.00 & 0.00\\ 0.00 & 2.6\times 10^{-5} & 0.00\\ 0.00 & 0.00 & 5\times 10^{-6} \end{array}\right]$ kg m^-2^				

### 4.2 Experimental comparison of improved prototype to original prototype

In order to validate and quantify the efficacy of the improvements we made, we conducted two experiments, one to measure the increase in AUJ angle range and another to measure the AUJ angle measurement accuracy. We also did not observe any string failure when $f_{\text{act}}\leq 9$ (no load limit monitoring failures), which demonstrated the efficacy of the improvements made to address that issue. Table 4 shows a summary of the effects of our improvements to the mechanism.

**TABLE 4:** Summary of all the performance improvements and the measurements used to quantify them.

Improvement	Measurement	Results	
		Original	Improved
Increasing AUJ angle range	Maximum AUJ angle	14.50°	26.00°
Improving AUJ angle measurement accuracy	Minimum step change in AUJ angle measurement	0.21°	0.10°
Preventing string failure	String failed when $f_{\text{act}}\leq 9$ ?	✓	✗

#### 4.2.1 AUJ angle range

**FIGURE 11:**
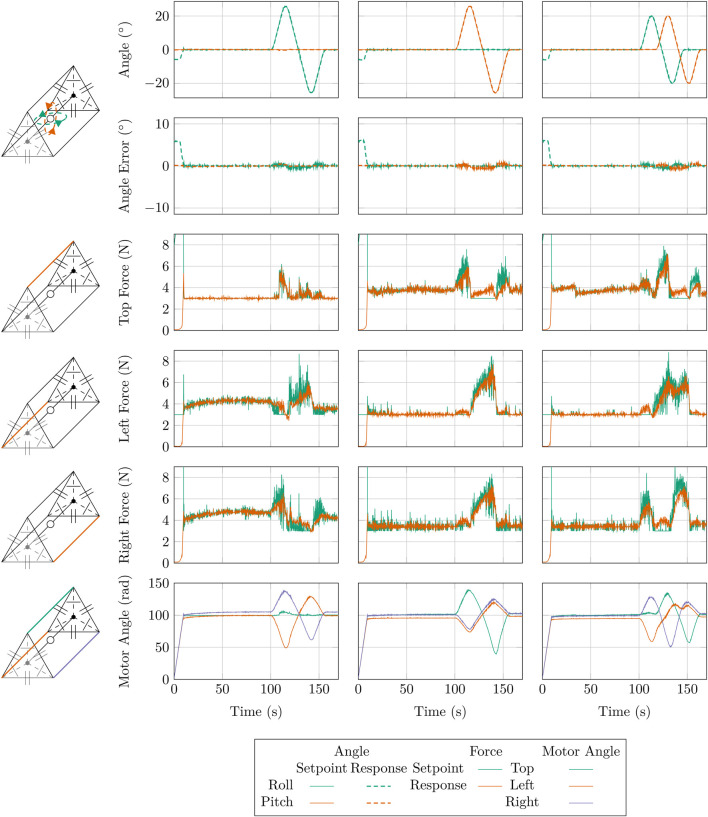
Plots of the response for three different trajectories, one on only the roll axis $\theta_{1}$ (column 1), one on only the pitch axis $\theta_{2}$ (column 2), and one on both axes $\theta_{1}$ and $\theta_{2}$ (column 3). Plots include AUJ orientation, forces at the top, left and right TSA, and the motor positions.

To evaluate the expanded AUJ angle range, we replicated the setpoint tracking experiments from Crosby et al. (2022), which consisted of a ‘‘pitch only’’, ‘‘roll only’’ and ‘‘pitch and roll’’ experiments. As with the previous experiments, each motor angle was limited to 250 rad, below the ‘‘twist limit’’ of 480.36413313235613 rad for each TSA as defined by Equation 2. Figure 11 illustrates the tracking response in both the pitch and roll axes. We used a similarly high $k_{p}$ value as in Crosby et al. (2022), which could be reduced if energy consumption is a issue, at the expense of increased tracking error. Mechanical limitations restricted the AUJ angle range to ± 26°. This constraint resulted from the hollow spider colliding with the TSA universal joints in the pitch axis and with the follower body in the roll axis. For the dual-axis trajectory, the angle range was further constrained to ± 20° due to a load cell exceeding the safety limit of 9 N (with the load cell full scale being 9.8 N (LCM100 Miniature In Line Load Cell, 2024). Future work will swap these load cells with load cells that have a larger full scale in order to remove this constraint.

#### 4.2.2 AUJ angle measurement accuracy

**FIGURE 12:**
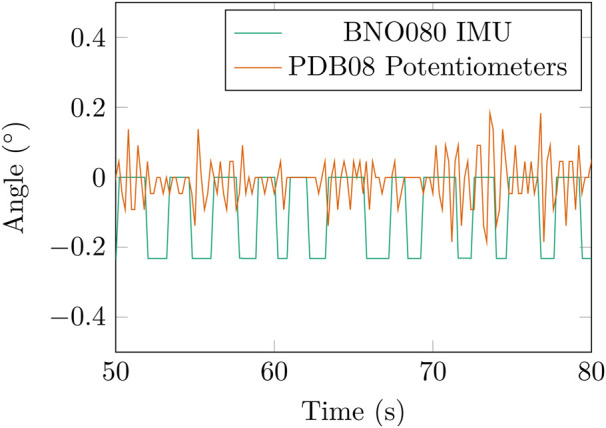
Comparison of the roll $\left(\theta_{1}\right)$ angle signal from the BNO080 IMU and PDB08 potentiometer when the AUJ is at rest at setpoint θ = [0 0].

Figure 12 shows the difference between the BNO080 IMU and PDB08 potentiometer when the AUJ is at rest. The minimum measured change in value was 0.21° for the BNO080 and 0.11° for the PDB08, a significant increase in measured accuracy.

## 5 Additional experimental results

### 5.1 The effect of follower mass on AUJ angle tracking

**FIGURE 13:**
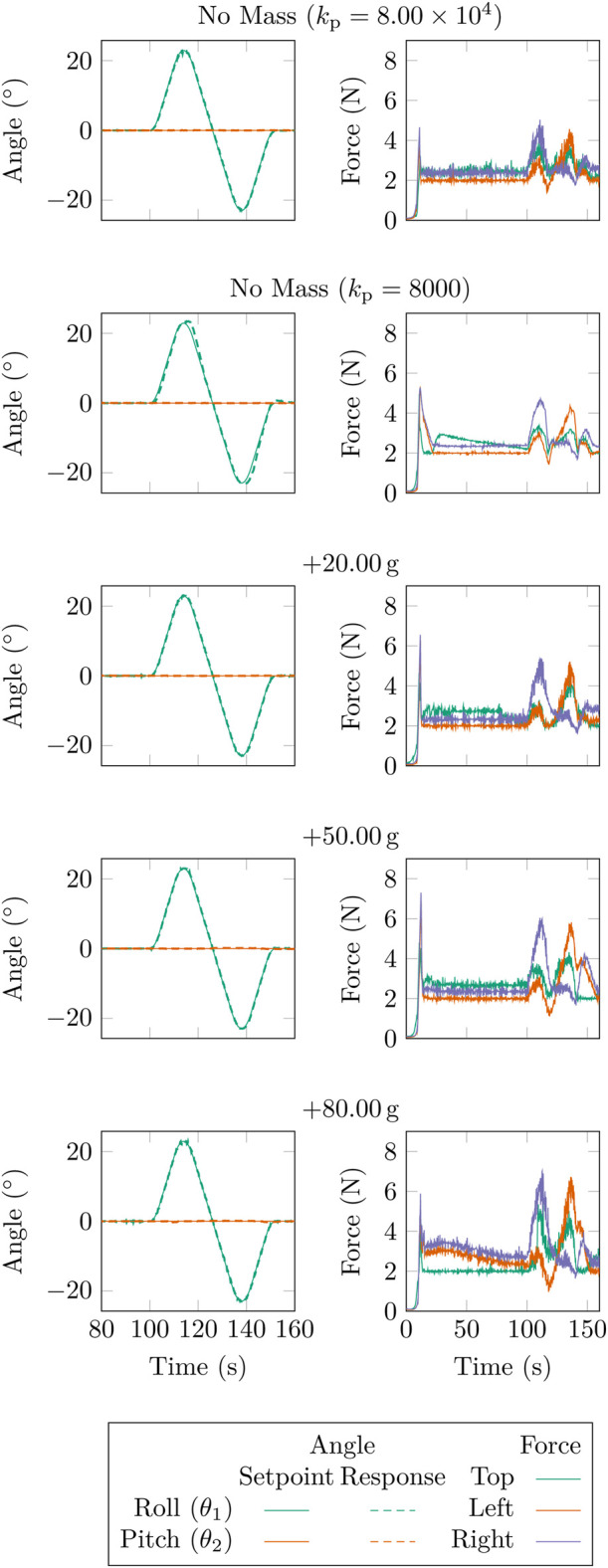
AUJ roll $\left(\theta_{1}\right)$ tracking with increasing follower mass from Table 5, plus with no mass at $k_{\text{p}}=8000$ and $k_{\text{p}}=8\times 10^{4}$ .

**FIGURE 14:**
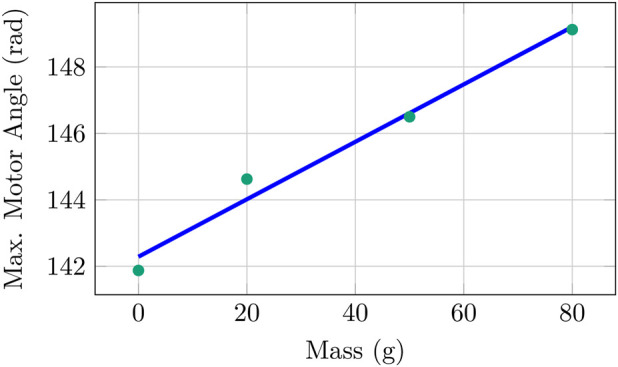
Maximum motor angle for increasing follower mass from Table 5. Note that $k_{p}$ for zero mass is $8\times 10^{4}$ .

For a multi-segment design, analysing the AUJ performance with a non-negligible follower mass is crucial. This addition alters the system dynamics, affecting the inverse dynamic calculations in the control system, as illustrated in Table 5. In Figure 13, the response for a roll trajectory with varying follower body masses (20 g, 50 g, and 80 g) is depicted. Due to the load cell safety limit of 9 N, larger masses could not be tested. Initially, setting $k_{\text{p}}$ to the value in Table 1 failed to achieve a steady state without significant oscillations except for the ‘‘No Mass’’ configuration, it is theorised that this is due to greater inertia from the additional mass. By tuning the $k_{\text{p}}$ gain to 8000 in weighted configurations, a steady state without significant oscillations was achieved with a maximum tracking error of 0.76°, similar to initial experiments. However, for the ‘‘No Mass’’ configuration, setting $k_{\text{p}}$ to 8000 resulted in a poor tracking error of 3.06°, contrasting with the 0.85° maximum tracking error obtained with $k_{\text{p}}$ from Table 3. This did reduce the load cell oscillations, as can be seen in Figure 13, which is expected for a lower value of $k_{\text{p}}$ and shows the oscillations are not due to sensor noise or structural resonance. This may suggest that $k_{\text{p}}$ will have to be further reduced as follower mass increases, in order to compensate for additional inertia. In future implementations, gain scheduling could optimise $k_{\text{p}}$ selection for a given follower mass, minimizing tracking error while ensuring a steady state. Increasing the follower mass also increased the maximum motor angle as shown in Figure 14 at a rate of ≈ 0.09 rad g^−1^. However, since there are only four data points it is unclear if this linear trend can be extrapolated.

**TABLE 5:** Table of all the follower mass configurations, with the parameters for follower mass m and follower COM z offset $\rho_{3}$ .

Config	m	$\rho_{3}$	I	Image
	[g]	[mm]	[kg m^-2^]	
No Mass	61.60	3.05	$\mathrm{diag}\left({\left[\begin{array}{c} 2.80\times 10^{-5}\\ 2.60\times 10^{-5}\\ 5.00\times 10^{-6} \end{array}\right]}^{⊺}\right)$	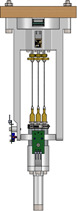
+20 g	81.29	15.74	$\mathrm{diag}\left({\left[\begin{array}{c} 6.90\times 10^{-5}\\ 6.80\times 10^{-5}\\ 7.00\times 10^{-6} \end{array}\right]}^{⊺}\right)$	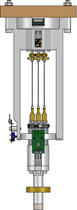
+50 g	111.10	26.39	$\mathrm{diag}\left({\left[\begin{array}{c} 1.06\times 10^{-4}\\ 1.05\times 10^{-4}\\ 1.10\times 10^{-5} \end{array}\right]}^{⊺}\right)$	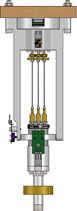
+80 g	140.35	32.44	$\mathrm{diag}\left({\left[\begin{array}{c} 1.29\times 10^{-4}\\ 1.27\times 10^{-4}\\ 1.20\times 10^{-5} \end{array}\right]}^{⊺}\right)$	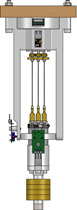

### 5.2 The effect of AUJ angular velocity on AUJ angle tracking

**FIGURE 15:**
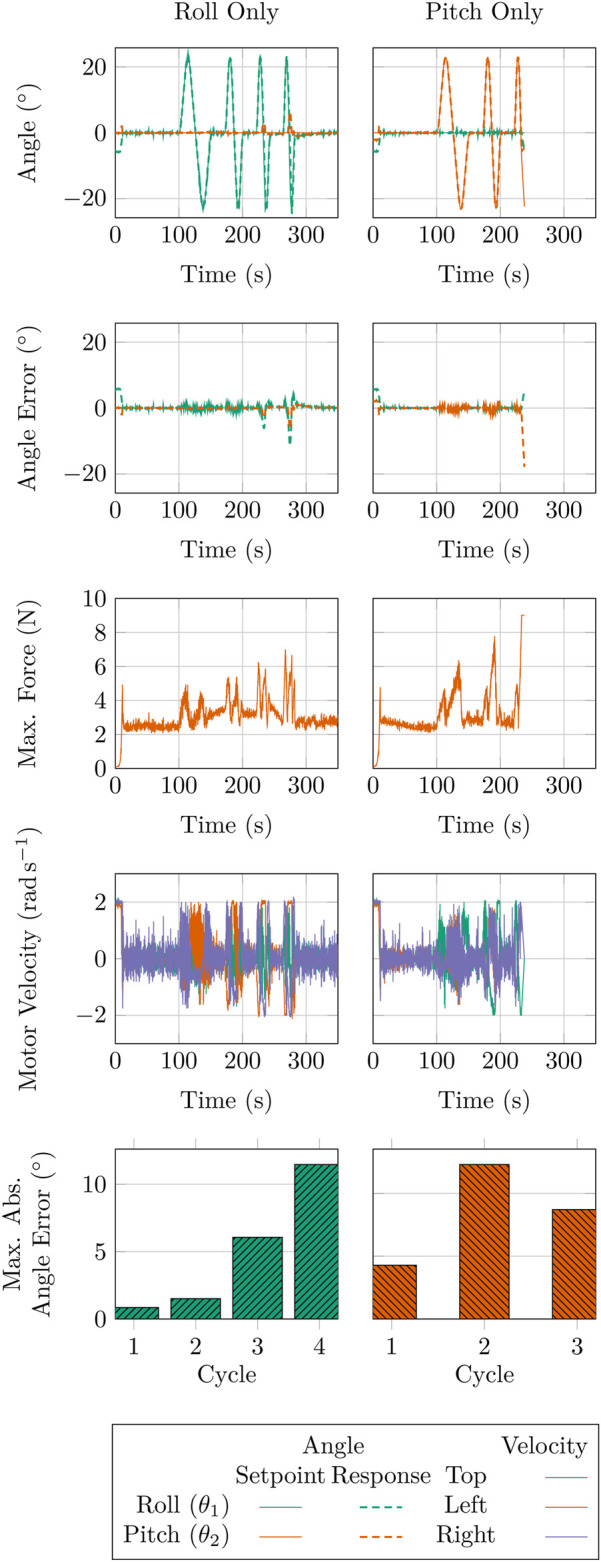
Results of the trapezoidal velocity trajectory from Table 6 for both AUJ pitch and roll trajectories, including the maximum absolute angle error for each cycle.

To assess the AUJ performance at higher angular velocities, we repeated the experiments from Section 4.2.1, but for each single axis, without any additional mass. Using a trapezoidal ‘‘chirp’’ signal, we reduced the angle range to ± 23° to prevent overshoot. Each cycle increased the maximum angular velocity by $\omega_{0}+\left(2\left(n-1\right)\omega_{0}\right)$ and the angular acceleration by $\alpha_{0}+\left(16\left(n-1\right)\alpha_{0}\right)$ , where n is the cycle number and $\omega_{0}$ and $\alpha_{0}$ are initial values. Four cycles were executed, with the maximum velocity and acceleration values detailed in Table 6. Figure 15 displays the experiment results, demonstrating a non-linear increase in tracking error with rising maximum angular velocity and acceleration. However, the pitch experiment could not be completed due to the load cell force exceeding the safety limit of 9 N, as evident in the figure.

**TABLE 6:** Trapezoidal trajectory sequence parameters.

Cycle	Max./Min. Angle	Max. Velocity	Acceleration
	[°]	[°s^-1^]	[°s^-2^]
1	22.92	22.92	0.57
2	22.92	25.21	0.63
3	22.92	27.73	0.69
4	22.92	30.50	0.76

## 6 Discussion

### 6.1 Active transmission adjustment (ATA) simulation

**FIGURE 16:**
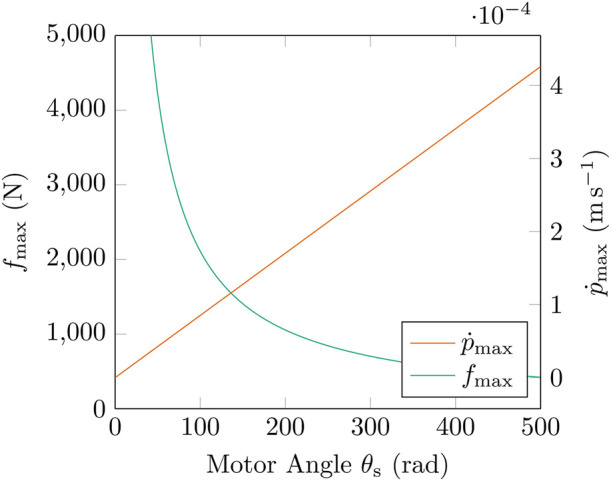
By adjusting $f_{\min}$ , the transmission ratio of the TSA can be altered. Reducing $f_{\min}$ increases the maximum TSA force $f_{\max}$ while reducing the maximum TSA stroke velocity ${\dot{p}}_{\max}$ . Conversely, increasing $f_{\min}$ reduces $f_{\max}$ and increases ${\dot{p}}_{\max}$ . This can be used to actively modify the dynamic properties of the AUJ during operation. In this graph, p=0 , and other coeffecients are from Table 3.

The TSA mechanism exhibits a non-linear transmission ratio dependent on $\theta_{\text{s}}$ (see Figure 16). This leads to an inverse relationship between the maximum stroke velocity ${\dot{p}}_{\max}$ and maximum tensile force $f_{\max}$ as $\theta_{\text{s}}$ increases. Using the jacobian J from Nedelchev et al. (2020), $f_{\max}$ and ${\dot{p}}_{\max}$ are found in Equation 5 as:

$$\begin{array}{cc} J & =\frac{\theta_{\text{s}}r_{\text{s}}^{2}}{l_{\text{u}}-p}\\ {J\;}^{-1} & =\frac{l_{\text{u}}-p}{\theta_{\text{s}}r_{\text{s}}^{2}}\\ f_{\max} & ={J\;}^{-1}\tau_{\max}\\ {\dot{p}}_{\max} & =J\dot{\theta }s_{\max}. \end{array}$$ (5)

Thus, increasing $\theta_{\text{s}}$ decreases $f_{\max}$ and increases ${\dot{p}}_{\max}$ for the same stroke p , with a greater effect if p increases. Adjusting $f_{\min}$ requires increasing the minimum value of $\theta_{\text{s}}$ for each TSA. This real-time alteration of $f_{\min}$ can modify TSA and AUJ performance during operation, allowing for maximum joint torque to be increased at the expense of joint velocity, or *vice versa*.

It may be noticed that $\lim_{\theta_{\text{s}}\to 0}f_{\max}=\infty$ , which is a consequence of assuming infinite material stiffness for the strings, which is not possible outside simulation. This poses a challenge for real-world high force applications, as discussed in Nedelchev et al. (2020).

**FIGURE 17:**
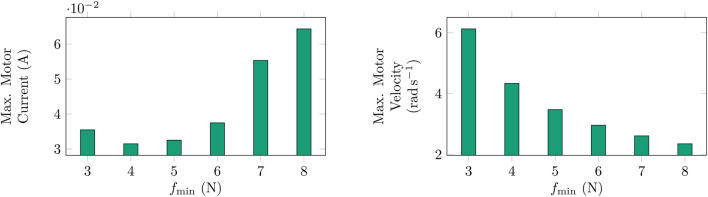
TSA maximum motor currents and velocities for a simulated roll $\left(\theta_{1}\right)$ trajectory from Figure 11 at different values of $f_{\min}$ . Simulation uses motor coefficients from Faulhaber 1724TSR motors (DC Micromotors - Precious Metal Commutation, 2020), as motor inertia and friction values are unavailable for Micro Metal Gearmotors. Note how the maximum motor current increases and the maximum motor velocity decreases as $f_{\min}$ increases for the same joint trajectory.

With the simulation of the mechanism used in Crosby et al. (2022) using the AUJ trajectory from Figure 11, a parameter sweep of $f_{\min}=\left\{3,4,5,6,7,8\right\}$ shows the expected increase in total motor torque $\sum_{i=1}^{3}\tau_{i}\left(t\right)$ and total TSA force $\sum_{i=1}^{3}f_{i}\left(t\right)$ for increasing values of $f_{\min}$ , as shown in Figure 17. Attempts were made to verify this experimentally with a modified parameter sweep to stay within load cell limits, however due to motor properties like gearbox backlash and PWM control nature, it was not possible to determine this relationship as the motor torque could not be measured accurately, and separate torque sensors were not available commercially at the scale required. Future work to enable experimental validation could explore better analysis methods or consider using brushless DC motors that may provide accurate enough torque sensing. Novel micro torque sensors using magnetostrictive materials (Muro et al., 2014) could also be utilised, or a larger prototype built to allow for the use of existing commercial micro torque sensors such as the FUTEK QTA141.

### 6.2 Dynamics gravity vector

In the experiments, the gravity vector g in the inverse dynamic control loop $C_{2}$ was assumed parallel to the z axis $\left(\left[\begin{array}{ccc} 0 & 0 & -9.81 \end{array}\right]\right)$ due to the vertical orientation of the mechanism. To function in other orientations, a IMU would be necessary to compute the gravity vector, incorporating gyroscopes to counter motion-generated forces.

### 6.3 Load cell limitation

As noted in Sections 4.2.1, 5.1 and 5.2, the load on each TSA being limited to 9.00 N meant that the AUJ angle range was limited with an increased follower mass, increased velocity, and in dual axis operation. Using load cells with a larger full scale would increase the AUJ angle range in all cases.

### 6.4 String lifecycle

Based on the results from Figure 11 we can observe that none of the motor angles exceed 139.8 rad, which is a stroke of 1.84 mm or 3.4% of $l_{\text{u}}$ . This gives a lifecycle of ≈ 51996 cycles using the curve fitting empirical model in Usman et al. (2017a), assuming a 5 kg load. However, as can be seen from Table 7, most comparable snake robots are significantly less than this mass, and we would expect a snake robot constructed with this mechanism to be even lighter considering the low mass of the TSA. Lifecycle can also be extended by material engineering, as in Sarkar et al. (2018). It must be noted that this is based on a model from existing experimental data and has not been experimentally replicated in this research. The performance of the controller may also be affected by fatigue effects as the number of cycles increase. This is something that may be considered for future work.

**TABLE 7:** List of snake robots, their number of segments n , their segment mass m , segment length l , maximum joint torque $\tau_{\max}$ , and self-supporting segment limit. Also includes three concept snake robots using the TSA AUJ mechanism, and three different values of $\theta_{\text{s}}$ .

Robot name	Image	n	m [kg]	l [mm]	$\tau_{\max}$ [N m]	$n_{\lim}$
Concept snake robot ( $\theta_{\text{s}}=$ 100.00rad)	-	-	0.30	155.00	392.23	41
Concept snake robot ( $\theta_{\text{s}}=$ 300.00rad)	-	-	0.30	155.00	130.74	23
Concept snake robot ( $\theta_{\text{s}}=$ 700.00rad)	-	-	0.30	155.00	56.03	15
Uncle sam Wright et al. (2012)	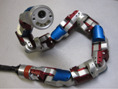	8	0.32	101.60	1.30	2
SEA snake Rollinson et al. (2014)	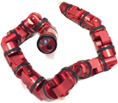	8	0.41	128.00	7.00	5
Zmeelok-3M Varlashin and Shmakov (2019)	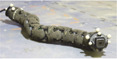	10	0.40	80.00	3.20	4
ACM-R5 Yamada (2005)	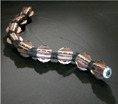	9	0.80	170.00	9.00	3
Wheeko Liljebäck et al. (2013)	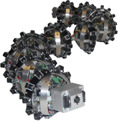	10	0.96	130.00	4.50	2

### 6.5 Multi-segment design

One potential practical application of this mechanism is to chain multiple TSA AUJ together to create a flexible robot comprised of multiple segments. Snake and snake-arm robots are common implementations of chains of AUJ where this mechanism could be implemented. Snake robots in particular are a promising application, since the actuators need to be inline unless the snake robot is tethered, and segment mass is an important factor for agility, manoeuvrability and accommodation of batteries and sensor packages.

#### 6.5.1 Self-supporting segment limit

If we consider a multi segment snake robot that consists of an arbitrary number of identical segments of mass m , length l and (COM) offset ρ along the z axis, the *self-supporting segment limit* can be defined as the maximum number of segments that can supported with no contact points under the influence of gravity g in any orientation. This is important for snake robots, as it determines how far they can reach when performing certain manoeuvres that require vertical traversal, such as climbing stairs. For snake-arm robots, it effectively is the limit for the number of segments, since all segments are continuously operating with no contact points. This limit $n_{\lim}\in N$ is defined by:

$$\begin{array}{cc} \tau_{\max} & \geq \overset{n-1}{\underset{i=0}{\sum }}mg\left(il+\rho \right),\\ \tau_{\max} & \geq mg\left(n\rho +\frac{ln\left(n-1\right)}{2}\right),\\ n_{\lim} & =\left\lfloor \frac{1}{2}-\frac{\rho -\sqrt{\frac{l^{2}}{4}-l\rho +\rho^{2}+\frac{2l\tau_{\max}}{mg}}}{l}\right\rfloor . \end{array}$$ (6)

which can be generalised to include designs with heterogeneous segments. Calculating $\tau_{\max}$ depends on the design of the actuation system. For ‘‘direct drive’’ motor driven systems, it is simply the maximum rated torque of the motor. For cable or linear actuator driven systems, it is $f_{\max}r$ , where $f_{\max}$ is the maximum rated force of the actuator, and r is the radius distance from the actuator to the pivot axis. For TSAs, this is extended to $\left(J^{-1}\tau_{\max}\right)r$ , as shown in Equation 5.

Table 7 gives a list of snake robots with inline actuators, identical segments, and their self-supporting segments limit. *Uncle Sam* and *SEA Snake* both have 16 segments of alternating 1 DOF joints, each pair of these is considered one segment in this analysis. Segment COM offsets are not provided, so it is assumed to be $\frac{l}{2}$ .

**FIGURE 18:**
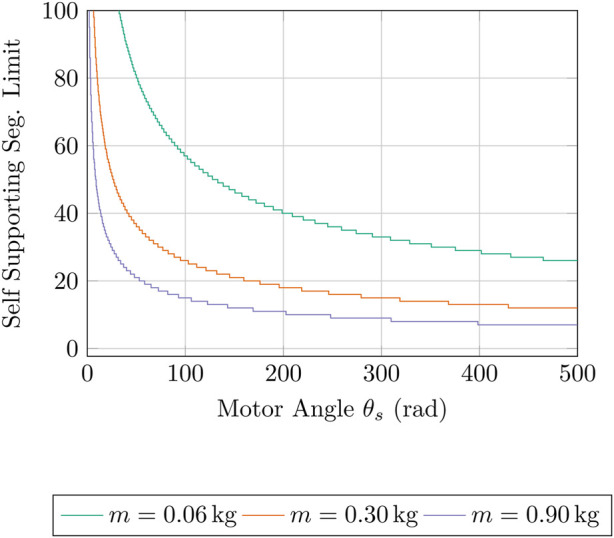
Graph showing the decrease in self-supporting segments limit as TSA motor angle $\theta_{\text{s}}$ increases for different segment masses m at p=0 . Segment length is 155 mm measured from the prototype, other coefficients as used in Figure 16 from Table 3.

We can then construct a theoretical snake robot made of the TSA AUJ segments in this publication, and see how it would compare to existing snake robots. However, since the prototype was constructed of lightweight 3D printed materials which are not designed to be durable, with a mass of only 61.6 g, a direct comparison would not be entirely fair. Therefore, we have considered designs with additional mass, weighing 300 g and 900 g respectively. This can represent a change in segment design, construction materials or additional components. Figure 18 shows the limit as $\theta_{\text{s}}$ increases, which outperforms all the robots in Table 7 within 150 rad, whereas the experiments conducted in Section 5 have a maximum motor angle of 139.8 rad, as shown in Figure 11. There are limitations to this analysis, as it uses a model for the TSA that does not take into account string compliance and friction, therefore physical implementations are not expected to perform as well as this simple geometric model. Using a more advanced dynamic model (Nedelchev et al., 2020) may provide more accurate results, but would require detailed knowledge or characterisation of the TSA string material properties and the mechanism as a whole, and is therefore proposed for future work. Most of the snake robots in Table 7 also have a larger joint angle range than the prototype design, which may be useful for some tasks, such as anchoring to a tree. However, it shows that the TSA AUJ mechanism has a lot of potential in developing highly mobile snake robots with many segments that are able to self-support the majority of said segments, which would allow for increased agility in many environments. It also allows for similarly long snake-arm robots with inline actuation.

#### 6.5.2 Distributed control system

**FIGURE 19:**
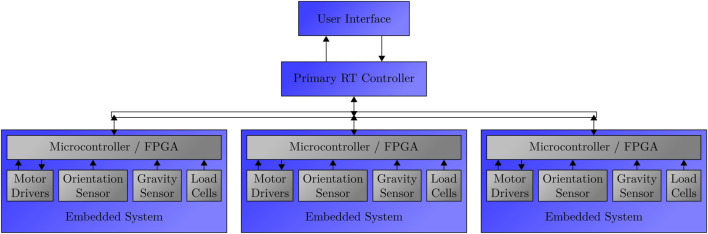
Proposed system architecture for a future multi-segment system. Each segment has an embedded controller programmed with the cascaded control loop. The controller interfaces with the load cells for each TSA, the orientation sensors for the AUJ, an accelerometer to provide the local gravity vector for dynamics calculations, and the drivers for the TSA motors. A primary controller then uses a common control bus to interface with the embedded controllers, reading and writing data to registers to issue motion commands and get status updates.

In a multi-segment setup, individual embedded control systems within each segment could receive AUJ angle position setpoints from a central controller. This setup enables the execution of complex trajectories while minimizing wiring complexity through shared power and communication buses, fostering a modular design. Figure 19 shows a conceptual system diagram for this setup.

## 7 Conclusion

This publication examines an existing prototype of a actuated universal joint with twisted string actuator to pinpoint enhancements and address limitations. Three key design improvements were identified and tested, resulting in an expanded AUJ angle range from ± 14.5° to ± 26°, increased AUJ measurement accuracy from 0.21° to 0.11°, and zero recorded string failures under normal conditions. Additionally, the impact of added follower mass on AUJ performance was assessed to simulate multi-segment operation. Future efforts will focus on resolving remaining limitations, including installing load cells with larger full scales, developing a distributed control system for multi-segment operation, and exploring effective methods for measuring motor current under (PWM) control or using brushless DC motors for accurate ATA assessment.

## Data Availability

The raw data supporting the conclusions of this article will be made available by the authors, without undue reservation.
